# Supplementary material for: A Meta-Analysis Study of SOD1-Mutant Mouse Models of ALS to Analyse the Determinants of Disease Onset and Progression
Source: Int J Mol Sci. 2022 Dec 22;24(1):216. doi: 10.3390/ijms24010216 (PMC9820332; doi:10.3390/ijms24010216)
Supplement: Supplementary file 1 [file ijms-24-00216-s001.zip › ijms-2075149-supplementary.pdf]

Reference list of primary studies included in metanalysis

1. Ahn, S.W. *et al*. Neuroprotective effects of JGK-263 in transgenic SOD1-G93A mice of amyotrophic lateral sclerosis. *J. Neurol. Sci.* **2014**, *340*, 112–116. DOI: 10.1016/j.jns.2014.03.008.
2. Ahn, S.W. *et al*. The neuroprotective effect of the GSK-3β inhibitor and influence on the extrinsic apoptosis in the ALS transgenic mice. *J. Neurol. Sci.* **2012**, *320*, 1–5. DOI: 10.1016/j.jns.2012.05.038.
3. Alves, C.J. *et al*. Early motor and electrophysiological changes in transgenic mouse model of amyotrophic lateral sclerosis and gender differences on clinical outcome. *Brain Res.* **2011**, *1394*, 90–104. DOI: 10.1016/j.brainres.2011.02.060.
4. Annelies, N. *et al*. Astrocyte-derived Jagged-1 mitigates deleterious Notch signaling in amyotrophic lateral sclerosis. *Neurobiol. Dis.* **2018**, *119*, 26–40. DOI: 10.1016/j.nbd.2018.07.012.
5. Apolloni, S. *et al*. Spinal cord pathology is ameliorated by P2X7 antagonism in a SOD1-mutant mouse model of amyotrophic lateral sclerosis. *DMM Dis. Model. Mech.* **2014**, *7*, 1101–1109. DOI: 10.1242/dmm.017038.
6. Apolloni, S. *et al*. Actions of the antihistaminergic clemastine on presymptomatic SOD1-G93A mice ameliorate ALS disease progression. *J. Neuroinflammation* **2016**, *13*, 191. DOI: 10.1186/s12974-016-0658-8.
7. Arrat, H. *et al*. ACTH (acthar gel) reduces toxic SOD1 protein linked to amyotrophic lateral sclerosis in transgenic mice: A novel observation. *PLoS One* **2015**, *10*, e125638. DOI: 10.1371/journal.pone.0125638.
8. Bai, L. *et al*. Simvastatin accelerated motoneurons death in SOD1G93A mice through inhibiting Rab7-mediated maturation of late autophagic vacuoles. *Cell Death Dis.* **2021**, *12*, 392. DOI: 10.1038/s41419-021-03669-w.
9. Banerjee, R. *et al*. Adaptive immune neuroprotection in G93A-SOD1 amyotrophic lateral sclerosis mice. *PLoS One* **2008**, *3*, e2740. DOI: 10.1371/journal.pone.0002740.
10. Benkler, C. *et al*. Multifactorial gene therapy enhancing the glutamate uptake system and reducing oxidative stress delays symptom onset and prolongs survival in the SOD1-G93A ALS mouse model. *J. Mol. Neurosci.* **2016**, *58*, 46–58. DOI: 10.1007/s12031-015-0695-2.
11. Biferi, M.G. *et al*. A new AAV10-U7-mediated gene therapy prolongs survival and restores function in an ALS mouse model. *Mol. Ther.* **2017**, *25*, 2038–2052. DOI: 10.1016/j.ymthe.2017.05.017.
12. Brambilla, L. *et al*. Disruption of the astrocytic TNFR1-GDNF axis accelerates motor neuron degeneration and disease progression in amyotrophic lateral sclerosis. *Hum. Mol. Genet.* **2016**, *25*, 3080–3095. DOI: 10.1093/hmg/ddw161.
13. Brenner, D. *et al*. Heterozygous Tbk1 loss has opposing effects in early and late stages of ALS in mice. *J. Exp. Med.* **2019**, *216*, 267–278. DOI: 10.1084/jem.20180729.
14. Buskila, Y. *et al*. Dynamic interplay between H-current and M-current controls motoneuron hyperexcitability in amyotrophic lateral sclerosis. *Cell Death Dis.* **2019**, *10*, 310. DOI: 10.1038/s41419-019-1538-9.
15. Calvo, A.C. *et al*. Lack of a synergistic effect of a non-viral ALS gene therapy based on BDNF and a TTC fusion molecule. *Orphanet J. Rare Dis.* **2011**, *6*, 10. DOI: 10.1186/1750-1172-6-10.
16. Chang-Hong, R. *et al*. Neuroprotective effect of oxidized galectin-1 in a transgenic mouse model of amyotrophic lateral sclerosis. *Exp. Neurol.* **2005**, *194*, 203–211. DOI: 10.1016/j.expneurol.2005.02.011.
17. Chiba, T. *et al*. Colivelin prolongs survival of an ALS model mouse. *Biochem. Biophys. Res. Commun.* **2006**, *343*, 793–798. DOI: 10.1016/j.bbrc.2006.02.184.
18. Chiu, I.M. *et al*. T lymphocytes potentiate endogenous neuroprotective inflammation in a mouse model of ALS. *Proc. Natl. Acad. Sci. U S A* **2008**, *105*, 17913–17918. DOI: 10.1073/pnas.0804610105.
19. Ciervo, Y. *et al*. Adipose-derived stem cells protect motor neurons and reduce glial activation in both in vitro and in vivo models of ALS. *Mol. Ther. Methods Clin. Dev.* **2021**, *21*, 413–433. DOI: 10.1016/j.omtm.2021.03.017.
20. Crosio, C. *et al*. Astroglial inhibition of NF-κb does not ameliorate disease onset and progression in a mouse model for amyotrophic lateral sclerosis (ALS). *PLoS One* **2011**, *6*, e17187. DOI: 10.1371/journal.pone.0017187.
21. Dobrowolny, G. *et al*. Muscle expression of a local Igf-1 isoform protects motor neurons in an ALS mouse model. *J. Cell Biol.* **2005**, *168*, 193–199. DOI: 10.1083/jcb.200407021.
22. Evans, T.M. *et al*. The effect of mild traumatic brain injury on peripheral nervous system pathology in wild-type mice and the G93A mutant mouse model of motor neuron disease. *Neuroscience* **2015**, *298*, 410–423. DOI: 10.1016/j.neuroscience.2015.04.041.
23. Evans, T.M. *et al*. Moderate modulation of disease in the G93A model of ALS by the compound 2-(2-hydroxyphenyl)-benzoxazole (HBX). *Neurosci. Lett.* **2016**, *624*, 1–7. DOI: 10.1016/j.neulet.2016.04.035.
24. Ezzi, S.A. *et al*. Neuronal over-expression of chromogranin A accelerates disease onset in a mouse model of ALS. *J. Neurochem.* **2010**, *115*, 1102–1111. DOI: 10.1111/j.1471-4159.2010.06979.x.
25. Fabbrizio, P. *et al*. P2X7 receptor activation modulates autophagy in SOD1-g93a mouse microglia. *Front. Cell. Neurosci.* **2017**, *11*, 249. DOI: 10.3389/fncel.2017.00249.

26. Feng, H.L. *et al*. Combined lithium and valproate treatment delays disease onset, reduces neurological deficits and prolongs survival in an amyotrophic lateral sclerosis mouse model. *Neuroscience* **2008**, *155*, 567–572. DOI: 10.1016/j.neuroscience.2008.06.040.
27. Garofalo, S. *et al*. Natural killer cells modulate motor neuron-immune cell cross talk in models of Amyotrophic Lateral Sclerosis. *Nat. Commun.* **2020**, *11*, 1773. DOI: 10.1038/s41467-020-15644-8.
28. Gerbino, V. *et al*. The loss of TBK1 kinase activity in motor neurons or in all cell types differentially impacts ALS disease progression in SOD1 mice. *Neuron* **2020**, *106*, 789-805.e5. DOI: 10.1016/j.neuron.2020.03.005.
29. Gianforcaro, A. and Hamadeh, M.J. Dietary vitamin D3 supplementation at 10× the adequate intake improves functional capacity in the G93A transgenic mouse model of ALS, a pilot study. *CNS Neurosci. Ther.* **2012**, *18*, 547–557. DOI: 10.1111/j.1755-5949.2012.00316.x.
30. Granucci, E.J. *et al*. Cromolyn sodium delays disease onset and is neuroprotective in the SOD1G93A Mouse Model of amyotrophic lateral sclerosis. *Sci. Rep.* **2019**, *9*, 17728. DOI: 10.1038/s41598-019-53982-w.
31. Gravel, M. *et al*. Il-10 controls early microglial phenotypes and disease onset in ALS caused by misfolded superoxide dismutase 1. *J. Neurosci.* **2016**, *36*, 1031–1048. DOI: 10.1523/JNEUROSCI.0854-15.2016.
32. Guerra, S. *et al*. Behavioural effects of cage systems on the G93A Superoxide Dismutase 1 transgenic mouse model for amyotrophic lateral sclerosis. *Genes Brain Behav.* **2021**, *20*, e12735. DOI: 10.1111/gbb.12735.
33. Guo, H. *et al*. Increased expression of the glial glutamate transporter EAAT2 modulates excitotoxicity and delays the onset but not the outcome of ALS in mice. *Hum. Mol. Genet.* **2003**, *12*, 2519–2532. DOI: 10.1093/hmg/ddg267.
34. Guo, Y. *et al*. The modest impact of transcription factor Nrf2 on the course of disease in an ALS animal model. *Lab. Invest.* **2013**, *93*, 825–833. DOI: 10.1038/labinvest.2013.73.
35. Hamadeh, M.J. *et al*. Caloric restriction transiently improves motor performance but hastens clinical onset of disease in the Cu/Zn-superoxide dismutase mutant G93A mouse. *Muscle Nerve* **2005**, *31*, 214–220. DOI: 10.1002/mus.20255.
36. Han, S. *et al*. Resveratrol upregulated heat shock proteins and extended the survival of G93A-SOD1 mice. *Brain Res.* **2012**, *1483*, 112–117. DOI: 10.1016/j.brainres.2012.09.022.
37. Han, Y. *et al*. Interleukin-6 deficiency does not affect motor neuron disease caused by superoxide dismutase 1 mutation. *PLoS One* **2016**, *11*, e0153399. DOI: 10.1371/journal.pone.0153399.
38. Hayworth, C.R. and Gonzalez-Lima, F. Pre-symptomatic detection of chronic motor deficits and genotype prediction in congenic B6.SOD1G93A ALS mouse model. *Neuroscience* **2009**, *164*, 975–985. DOI: 10.1016/j.neuroscience.2009.08.031.
39. Henriques, A. *et al*. Characterization of a novel SOD-1(G93A) transgenic mouse line with very decelerated disease development. *PLoS One* **2010**, *5*, e15445. DOI: 10.1371/journal.pone.0015445.
40. Ilieva, H.S. *et al*. Mutant dynein (Loa) triggers proprioceptive axon loss that extends survival only in the SOD1 ALS model with highest motor neuron death. *Proc. Natl. Acad. Sci. U S A* **2008**, *105*, 12599–12604. DOI: 10.1073/pnas.0805422105.
41. Israelson, A. *et al*. Misfolded mutant SOD1 directly inhibits VDAC1 conductance in a mouse model of inherited ALS. *Neuron* **2010**, *67*, 575–587. DOI: 10.1016/j.neuron.2010.07.019.
42. Jeon, G.S. *et al*. Neuroprotective effect of human adipose stem cell-derived extract in amyotrophic lateral sclerosis. *Neurochem. Res.* **2016**, *41*, 913–923. DOI: 10.1007/s11064-015-1774-z.
43. Jiang, H.Q. *et al*. Guanabenz delays the onset of disease symptoms, extends lifespan, improves motor performance and attenuates motor neuron loss in the SOD1 G93A mouse model of amyotrophic lateral sclerosis. *Neuroscience* **2014**, *277*, 132–138. DOI: 10.1016/j.neuroscience.2014.03.047.
44. Kang, J. and Rivest, S. MyD88-deficient bone marrow cells accelerate onset and reduce survival in a mouse model of amyotrophic lateral sclerosis. *J. Cell Biol.* **2007**, *179*, 1219–1230. DOI: 10.1083/jcb.200705046.
45. Kang, S.H. *et al*. Degeneration and impaired regeneration of gray matter oligodendrocytes in amyotrophic lateral sclerosis. *Nat. Neurosci.* **2013**, *16*, 571–579. DOI: 10.1038/nn.3357.
46. Kiaei, M. *et al*. Celastrol blocks neuronal cell death and extends life in transgenic mouse model of amyotrophic lateral sclerosis. *Neurodegener. Dis.* **2006**, *2*, 246–254. DOI: 10.1159/000090364.
47. Kira, Y. *et al*. L-Carnitine suppresses the onset of neuromuscular degeneration and increases the life span of mice with familial amyotrophic lateral sclerosis. *Brain Res.* **2006**, *1070*, 206–214. DOI: 10.1016/j.brainres.2005.11.052.
48. Koh, S.H. *et al*. Inhibition of glycogen synthase kinase-3 suppresses the onset of symptoms and disease progression of G93A-SOD1 mouse model of ALS. *Exp. Neurol.* **2007**, *205*, 336–346. DOI: 10.1016/j.expneurol.2007.03.004.
49. Koh, S.H. *et al*. Recombinant human erythropoietin suppresses symptom onset and progression of G93A-SOD1 mouse model of ALS by preventing motor neuron death and inflammation. *Eur. J. Neurosci.* **2007**, *25*, 1923–1930. DOI: 10.1111/j.1460-9568.2007.05471.x.
50. Koh, S.H. *et al*. The effect of epigallocatechin gallate on suppressing disease progression of ALS model mice. *Neurosci. Lett.* **2006**, *395*, 103–107. DOI: 10.1016/j.neulet.2005.10.056.

51. Korhonen, P. *et al*. Long-term interleukin-33 treatment delays disease onset and alleviates astrocytic activation in a transgenic mouse model of amyotrophic lateral sclerosis. *IBRO Rep.* **2019**, *6*, 74–86. DOI: 10.1016/j.ibror.2019.01.005.
52. Krishnan, J. *et al*. Over-expression of Hsp27 does not influence disease in the mutant SOD1 G93A mouse model of amyotrophic lateral sclerosis. *J. Neurochem.* **2008**, *106*, 2170–2183. DOI: 10.1111/j.1471-4159.2008.05545.x.
53. Kupershmidt, L. *et al*. Neuroprotective and neuritogenic activities of novel multimodal iron-chelating drugs in motor-neuron-like NSC-34 cells and transgenic mouse model of amyotrophic lateral sclerosis. *FASEB J.* **2009**, *23*, 3766–3779. DOI: 10.1096/fj.09-130047.
54. Lee, J. K. *et al*. Iron accumulation promotes TACE-mediated TNF-α secretion and neurodegeneration in a mouse model of ALS. *Neurobiol. Dis.* **2015**, *80*, 63–69. DOI: 10.1016/j.nbd.2015.05.009.
55. Lee, J.K. *et al*. MST1 functions as a key modulator of neurodegeneration in a mouse model of ALS. *Proc. Natl. Acad. Sci. U S A* **2013**, *110*, 12066–12071. DOI: 10.1073/pnas.1300894110.
56. Lee, S. *et al*. Activation of HIPK2 promotes ER stress-mediated neurodegeneration in amyotrophic lateral sclerosis. *Neuron* **2016**, *91*, 41–55. DOI: 10.1016/j.neuron.2016.05.021.
57. Lemarchant, S. *et al*. ADAMTS-4 promotes neurodegeneration in a mouse model of amyotrophic lateral sclerosis. *Mol. Neurodegener.* **2016**, *11*, 10. DOI: 10.1186/s13024-016-0078-3.
58. Lepore, A.C. *et al*. Intraparenchymal spinal cord delivery of adeno-associated virus IGF-1 is protective in the SOD1G93A model of ALS. *Brain Res.* **2007**, *1185*, 256–265. DOI: 10.1016/j.brainres.2007.09.034.
59. Lever, T.E. *et al*. An animal model of oral dysphagia in amyotrophic lateral sclerosis. *Dysphagia* **2009**, *24*, 180–195. DOI: 10.1007/s00455-008-9190-z.
60. Lewandowski, S.A. *et al*. Presymptomatic activation of the PDGF-CC pathway accelerates onset of ALS neurodegeneration. *Acta Neuropathol.* **2016**, *131*, 453–464. DOI: 10.1007/s00401-015-1520-2.
61. Leyton-Jaimes, M.F. *et al*. Endogenous macrophage migration inhibitory factor reduces the accumulation and toxicity of misfolded SOD1 in a mouse model of ALS. *Proc. Natl. Acad. Sci. U S A* **2016**, *113*, 10198–10203. DOI: 10.1073/pnas.1604600113.
62. Leyton-Jaimes, M.F. *et al*. AAV2/9-mediated overexpression of MIF inhibits SOD1 misfolding, delays disease onset, and extends survival in mouse models of ALS. *Proc. Natl. Acad. Sci. U S A* **2019**, *116*, 14755–14760. DOI: 10.1073/pnas.1904665116.
63. Li, W. *et al*. N-acetyl-l-tryptophan delays disease onset and extends survival in an amyotrophic lateral sclerosis transgenic mouse model. *Neurobiol. Dis.* **2015**, *80*, 93–103. DOI: 10.1016/j.nbd.2015.05.002.
64. Li, Y. *et al*. Trehalose decreases mutant SOD1 expression and alleviates motor deficiency in early but not end-stage amyotrophic lateral sclerosis in a SOD1-G93A mouse model. *Neuroscience* **2015**, *298*, 12–25. DOI: 10.1016/j.neuroscience.2015.03.061.
65. Liebl, M.P. *et al*. Low-frequency magnetic fields do not aggravate disease in mouse models of Alzheimer’s disease and amyotrophic lateral sclerosis. *Sci. Rep.* **2015**, *5*, 8585. DOI: 10.1038/srep08585.
66. Lin, H.Q. *et al*. Intramuscular delivery of scAAV9-hIGF1 prolongs survival in the hSOD1G93A ALS mouse model via upregulation of D-amino acid oxidase. *Mol. Neurobiol.* **2018**, *55*, 682–695. DOI: 10.1007/s12035-016-0335-z.
67. Lin, P.Y. *et al*. Heat shock factor 1 over-expression protects against exposure of hydrophobic residues on mutant SOD1 and early mortality in a mouse model of amyotrophic lateral sclerosis. *Mol. Neurodegener.* **2013**, *8*, 43. DOI: 10.1186/1750-1326-8-43.
68. Ling, K.K. *et al*. Antisense-mediated reduction of EphA4 in the adult CNS does not improve the function of mice with amyotrophic lateral sclerosis. *Neurobiol. Dis.* **2018**, *114*, 174–183. DOI: 10.1016/j.nbd.2018.03.002.
69. Liu, H.N. *et al*. Targeting of monomer/misfolded SOD1 as a therapeutic strategy for amyotrophic lateral sclerosis. *J. Neurosci.* **2012**, *32*, 8791–8799. DOI: 10.1523/JNEUROSCI.5053-11.2012.
70. Liu, L. *et al*. Effects of RAGE inhibition on the progression of the disease in hSOD1G93A ALS mice. *Pharmacol. Res. Perspect.* **2020**, *8*, e00636. DOI: 10.1002/prp2.636.
71. Lobsiger, C.S. *et al*. Schwann cells expressing dismutase active mutant SOD1 unexpectedly slow disease progression in ALS mice. *Proc. Natl. Acad. Sci. U S A* **2009**, *106*, 4465–4470. DOI: 10.1073/pnas.0813339106.
72. Maier, M. *et al*. A human-derived antibody targets misfolded SOD1 and ameliorates motor symptoms in mouse models of amyotrophic lateral sclerosis. *Sci. Transl. Med.* **2018**, *10,* eaah3924. DOI: 10.1126/scitranslmed.aah3924.
73. Marino, M. *et al*. Differences in protein quality control correlate with phenotype variability in 2 mouse models of familial amyotrophic lateral sclerosis. *Neurobiol. Aging* **2015**, *36*, 492–504. DOI: 10.1016/j.neurobiolaging.2014.06.026.
74. McLeod, V.M. *et al*. Androgen receptor antagonism accelerates disease onset in the SOD1G93A mouse model of amyotrophic lateral sclerosis. *Br. J. Pharmacol.* **2019**, *176*, 2111–2130. DOI: 10.1111/bph.14657.
75. Mesci, P. *et al*. System xC- is a mediator of microglial function and its deletion slows symptoms in amyotrophic lateral sclerosis mice. *Brain* **2015**, *138*, 53–68. DOI: 10.1093/brain/awu312.

76. Mitsui, S. *et al*. Systemic overexpression of SQSTM1/p62 accelerates disease onset in a SOD1H46R-expressing ALS mouse model. *Mol. Brain* **2018**, *11*, 30. DOI: 10.1186/s13041-018-0373-8.
77. Mòdol-Caballero, G. *et al*. Gene therapy for overexpressing Neuregulin 1 type I in skeletal muscles promotes functional improvement in the SOD1G93A ALS mice. *Neurobiol. Dis.* **2020**, *137*, 104793. DOI: 10.1016/j.nbd.2020.104793.
78. Morrison, B.M. *et al*. A soluble activin type IIB receptor improves function in a mouse model of amyotrophic lateral sclerosis. *Exp. Neurol.* **2009**, *217*, 258–268. DOI: 10.1016/j.expneurol.2009.02.017.
79. Nagano, S. *et al*. A cysteine residue affects the conformational state and neuronal toxicity of mutant SOD1 in mice: Relevance to the pathogenesis of ALS. *Hum. Mol. Genet.* **2015**, *24*, 3427–3439. DOI: 10.1093/hmg/ddv093.
80. Nardo, G. *et al*. Counteracting roles of MHCI and CD8+ T cells in the peripheral and central nervous system of ALS SOD1G93A mice. *Mol. Neurodegener.* **2018**, *13*, 42. DOI: 10.1186/s13024-018-0271-7.
81. Ono, Y. *et al*. SA4503, a sigma-1 receptor agonist, suppresses motor neuron damage in in vitro and in vivo amyotrophic lateral sclerosis models. *Neurosci. Lett.* **2014**, *559*, 174–178. DOI: 10.1016/j.neulet.2013.12.005.
82. Pasquarelli, N. *et al*. Evaluation of monoacylglycerol lipase as a therapeutic target in a transgenic mouse model of ALS. *Neuropharmacology* **2017**, *124*, 157–169. DOI: 10.1016/j.neuropharm.2017.03.037.
83. Patel, B.P. *et al*. Caloric restriction shortens lifespan through an increase in lipid peroxidation, inflammation and apoptosis in the G93A mouse, an animal model of ALS. *PLoS One* **2010**, *5*, e9386. DOI: 10.1371/journal.pone.0009386.
84. Pfohl, S.R. *et al*. Characterization of the contribution of genetic background and gender to disease progression in the SOD1 G93A mouse model of amyotrophic lateral sclerosis: a meta-analysis. *J. Neuromuscul. Dis.* **2015**, *2*, 137–150. DOI: 10.3233/JND-140068.
85. Potenza, R.L. *et al*. Effects of chronic caffeine intake in a mouse model of amyotrophic lateral sclerosis. *J. Neurosci. Res.* **2013**, *91*, 585–592. DOI: 10.1002/jnr.23185.
86. Potenza, R.L. *et al*. Fingolimod: a disease-modifier drug in a mouse model of amyotrophic lateral sclerosis. *Neurotherapeutics* **2016**, *13*, 918–927. DOI: 10.1007/s13311-016-0462-2.
87. Pozzi, S. *et al*. Chronic administration of pimozide fails to attenuate motor and pathological deficits in two mouse models of amyotrophic lateral sclerosis. *Neurotherapeutics* **2018**, *15*, 715–727. DOI: 10.1007/s13311-018-0634-3.
88. Puigdomenech-Poch, M. *et al*. Dual role of lysophosphatidic acid receptor 2 (LPA2) in amyotrophic lateral sclerosis. *Front. Cell. Neurosci.* **2021**, *15*, 600872. DOI: 10.3389/fncel.2021.600872.
89. Ralph, G.S. *et al*. Silencing mutant SOD1 using RNAi protects against neurodegeneration and extends survival in an ALS model. *Nat. Med.* **2005**, *11*, 429–433. DOI: 10.1038/nm1205.
90. Rando, A. *et al*. Chemotherapeutic agent 5-fluorouracil increases survival of SOD1 mouse model of ALS. *PLoS One* **2019**, *14*, e0210752. DOI: 10.1371/journal.pone.0210752.
91. Raoul, C. *et al*. Lentiviral-mediated silencing of SOD1 through RNA interference retards disease onset and progression in a mouse model of ALS. *Nat. Med.* **2005**, *11*, 423–428. DOI: 10.1038/nm1207.
92. Riehm, J.J. *et al*. Poloxamer 188 decreases membrane toxicity of mutant SOD1 and ameliorates pathology observed in SOD1 mouse model for ALS. *Neurobiol. Dis.* **2018**, *115*, 115–126. DOI: 10.1016/j.nbd.2018.03.014.
93. Rosenblum, L.T. *et al*. Mutation of the caspase-3 cleavage site in the astroglial glutamate transporter EAAT2 delays disease progression and extends lifespan in the SOD1-G93A mouse model of ALS. *Exp. Neurol.* **2017**, *292*, 145–153. DOI: 10.1016/j.expneurol.2017.03.014.
94. Ross, E.K. *et al*. A cystine-rich whey supplement (Immunocal®) delays disease onset and prevents spinal cord glutathione depletion in the hSOD1G93A mouse model of amyotrophic lateral sclerosis. *Antioxidants* **2014**, *3*, 843–865. DOI: 10.3390/antiox3040843.
95. Rudnick, N.D. *et al*. Distinct roles for motor neuron autophagy early and late in the SOD1G93A mouse model of ALS. *Proc. Natl. Acad. Sci. U S A* **2017**, *114*, E8294–E8303. DOI: 10.1073/pnas.1704294114.
96. Rué, L. *et al*. Reduction of ephrin-A5 aggravates disease progression in amyotrophic lateral sclerosis. *Acta Neuropathol. Commun.* **2019**, *7*(1), 114. DOI: 10.1186/s40478-019-0759-6.
97. Ruiz-Ruiz, C. *et al*. Chronic administration of P2X7 receptor antagonist JNJ-47965567 delays disease onset and progression, and improves motor performance in ALS SOD1G93A female mice. *Dis. Model. Mech.* **2020**, *13*, dmm045732. DOI: 10.1242/dmm.045732.
98. Scekic-Zahirovic, J. *et al*. Evidence that corticofugal propagation of ALS pathology is not mediated by prion-like mechanism. *Prog. Neurobiol.* **2021**, *200*, 101972. DOI: 10.1016/j.pneurobio.2020.101972.
99. Schiaffino, L. *et al*. Acetylation state of RelA modulated by epigenetic drugs prolongs survival and induces a neuroprotective effect on ALS murine model. *Sci. Rep.* **2018**, *8*, 12875. DOI: 10.1038/s41598-018-30659-4.
100. Seijffers, R. *et al*. ATF3 expression improves motor function in the ALS mouse model by promoting motor neuron survival and retaining muscle innervation. *Proc. Natl. Acad. Sci. U S A* **2014**, *111*, 1622–1627. DOI: 10.1073/pnas.1314826111.

101. Shang, H.Y. *et al*. Therapeutic effects of hirsutella sinensis on the disease onset and progression of amyotrophic lateral sclerosis in SOD1G93A transgenic mouse model. *CNS Neurosci. Ther.* **2020**, *26*, 90–100. DOI: 10.1111/cns.13182.
102. Shimojo, Y. *et al*. Effect of rosmarinic acid in motor dysfunction and life span in a mouse model of familial amyotrophic lateral sclerosis. *J. Neurosci. Res.* **2010**, *88*, 896–904. DOI: 10.1002/jnr.22242.
103. Soon, C.P.W. *et al*. Diacetylbis(N(4)-methylthiosemicarbazonato) copper(II) (Cu II(atsm)) protects against peroxynitrite-induced nitrosative damage and prolongs survival in amyotrophic lateral sclerosis mouse model. *J. Biol. Chem.* **2011**, *286*, 44035–44044. DOI: 10.1074/jbc.M111.274407.
104. Sun, H. *et al*. Multiple systemic transplantations of human amniotic mesenchymal stem cells exert therapeutic effects in an ALS mouse model. *Cell Tissue Res.* **2014**, *357*, 571–582. DOI: 10.1007/s00441-014-1903-z.
105. Taes, I. *et al*. Tau levels do not influence human ALS or motor neuron degeneration in the SOD1G93A mouse. *Neurology* **2010**, *74*, 1687–1693. DOI: 10.1212/WNL.0b013e3181e042f7.
106. Tan, H. *et al*. LanCL1 promotes motor neuron survival and extends the lifespan of amyotrophic lateral sclerosis mice. *Cell Death Differ.* **2020**, *27*, 1369–1382. DOI: 10.1038/s41418-019-0422-6.
107. Tanaka, K. *et al*. A dopamine receptor antagonist L-745,870 suppresses microglia activation in spinal cord and mitigates the progression in ALS model mice. *Exp. Neurol.* **2008**, *211*, 378–386. DOI: 10.1016/j.expneurol.2008.02.004.
108. Thomsen, G.M. *et al*. Delayed disease onset and extended survival in the SOD1G93A rat model of amyotrophic lateral sclerosis after suppression of mutant SOD1 in the motor cortex. *J. Neurosci.* **2014**, *34*, 15587–15600. https://doi.org/10.1523/JNEUROSCI.2037-14.2014.
109. Tokuda, E. *et al*. Overexpression of metallothionein-I, a copper-regulating protein, attenuates intracellular copper dyshomeostasis and extends lifespan in a mouse model of amyotrophic lateral sclerosis caused by mutant superoxide dismutase-1. *Hum. Mol. Genet.* **2014**, *23*, 1271–1285. DOI: 10.1093/hmg/ddt517.
110. Tremblay, E. *et al*. Opposite synaptic alterations at the neuromuscular junction in an ALS mouse model: when motor units matter. *J. Neurosci.* **2017**, *37*, 8901–8918. DOI: 10.1523/JNEUROSCI.3090-16.2017.
111. Tung, Y.T. *et al*. Mir-17~92 confers motor neuron subtype differential resistance to ALS-associated degeneration. *Cell Stem Cell* **2019**, *25*, 193-209.e7. DOI: 10.1016/j.stem.2019.04.016.
112. Urushitani, M. *et al*. Therapeutic effects of immunization with mutant superoxide dismutase in mice models of amyotrophic lateral sclerosis. *Proc. Natl. Acad. Sci. U S A* **2007**, *104*, 2495–2500. DOI: 10.1073/pnas.0606201104.
113. Vallarola, A. *et al*. RNS60 exerts therapeutic effects in the SOD1 ALS mouse model through protective glia and peripheral nerve rescue. *J. Neuroinflammation* **2018**, *15*, 65. DOI: 10.1186/s12974-018-1101-0.
114. Vallarola, A. *et al*. A novel HGF/SF receptor (MET) agonist transiently delays the disease progression in an amyotrophic lateral sclerosis mouse model by promoting neuronal survival and dampening the immune dysregulation. *Int. J. Mol. Sci.* **2020**, *21*, 8542. DOI: 10.3390/ijms21228542.
115. Venturin, G.T. *et al*. Transplantation of bone marrow mononuclear cells prolongs survival, delays disease onset and progression and mitigates neuronal loss in pre-symptomatic, but not symptomatic ALS mice. *Neurosci. Lett.* **2016**, *633*, 182–188. DOI: 10.1016/j.neulet.2016.09.030.
116. Wang, L. *et al*. Wild-type SOD1 overexpression accelerates disease onset of a G85R SOD1 mouse. *Hum. Mol. Genet.* **2009**, *18*, 1642–1651. DOI: 10.1093/hmg/ddp085.
117. Wang, L. *et al*. Mutant SOD1 knockdown in all cell types ameliorates disease in G85R SOD1 mice with a limited additional effect over knockdown restricted to motor neurons. *J. Neurochem.* **2010**, *113*, 166–174. DOI: 10.1111/j.1471-4159.2010.06594.x.
118. Wang, L. *et al*. Astrocyte loss of mutant SOD1 delays ALS disease onset and progression in G85R transgenic mice. *Hum. Mol. Genet.* **2011**, *20*, 286–293. DOI: 10.1093/hmg/ddq463.
119. Wang, L. *et al*. The unfolded protein response in familial amyotrophic lateral sclerosis. *Hum. Mol. Genet.* **2011**, *20*, 1008–1015. DOI: 10.1093/hmg/ddq546.
120. Wang, L. *et al*. An enhanced integrated stress response ameliorates mutant SOD1-induced ALS. *Hum. Mol. Genet.* **2014**, *23*, 2629–2638. DOI: 10.1093/hmg/ddt658.
121. Wang, L. *et al*. Guanabenz, which enhances the unfolded protein response, ameliorates mutant SOD1-induced amyotrophic lateral sclerosis. *Neurobiol. Dis.* **2014**, *71*, 317–324. DOI: 10.1016/j.nbd.2014.08.010.
122. Wang, L. *et al*. Selective knockdown of mutant SOD1 in Schwann cells ameliorates disease in G85R mutant SOD1 transgenic mice. *Neurobiol. Dis.* **2012**, *48*, 52–57. DOI: 10.1016/j.nbd.2012.05.014.
123. Wang, L. *et al*. The effect of mutant SOD1 dismutase activity on non-cell autonomous degeneration in familial amyotrophic lateral sclerosis. *Neurobiol. Dis.* **2009**, *35*, 234–240. DOI: 10.1016/j.nbd.2009.05.002.
124. Williamson, T.L. *et al*. Absence of neurofilaments reduces the selective vulnerability of motor neurons and slows disease caused by a familial amyotrophic lateral sclerosis-linked superoxide dismutase 1 mutant. *Proc. Natl. Acad. Sci. U S A* **1998**, *95*, 9631–9636. DOI: 10.1073/pnas.95.16.9631.

125. Winkler, E.A. *et al*. Blood-spinal cord barrier disruption contributes to early motor-neuron degeneration in ALS-model mice. *Proc. Natl. Acad. Sci. U S A* **2014**, *111*, E1035-E1042. DOI: 10.1073/pnas.1401595111.
126. Winter, A.N. *et al*. An anthocyanin-enriched extract from strawberries delays disease onset and extends survival in the hSOD1G93A mouse model of amyotrophic lateral sclerosis. *Nutr. Neurosci.* **2018**, *21*, 414–426. DOI: 10.1080/1028415X.2017.1297023.
127. Zeldich, E. *et al*. Klotho is neuroprotective in the superoxide dismutase (SOD1G93A) mouse model of ALS. *J. Mol. Neurosci.* **2019**, *69*, 264–285. DOI: 10.1007/s12031-019-01356-2.
128. Zhang, J.J. *et al*. Repurposing carbamazepine for the treatment of amyotrophic lateral sclerosis in SOD1-G93A mouse model. *CNS Neurosci. Ther.* **2018**, *24*, 1163–1174. DOI: 10.1111/cns.12855.
129. Zhang, X. *et al*. Verapamil ameliorates motor neuron degeneration and improves lifespan in the SOD1G93A mouse model of ALS by enhancing autophagic flux. *Aging Dis.* **2019**, *10*, 1159–1173. DOI: 10.14336/AD.2019.0228.
130. Zhang, X. *et al*. MTOR-independent, autophagic enhancer trehalose prolongs motor neuron survival and ameliorates the autophagic flux defect in a mouse model of amyotrophic lateral sclerosis. *Autophagy* **2014**, *10*, 588–602. DOI: 10.4161/auto.27710.
131. Zhang, Y. *et al*. Treatment with hydrogen-rich saline delays disease progression in a mouse model of amyotrophic lateral sclerosis. *Neurochem. Res.* **2016**, *41*, 770–778. DOI: 10.1007/s11064-015-1750-7.
132. Zhao, B. *et al*. Therapeutic vaccines for amyotrophic lateral sclerosis directed against disease specific epitopes of superoxide dismutase 1. *Vaccine*, **2019**, *37*, 4920–4927. DOI: 10.1016/j.vaccine.2019.07.044.
133. Zhao, J. *et al*. Decreased signalling of EphA4 improves functional performance and motor neuron survival in the SOD1G93A ALS mouse model. *Sci. Rep.* **2018**, *8*, 11393. DOI: 10.1038/s41598-018-29845-1.
134. Zhao, Z. *et al*. Neuroprotective effects of genistein in a SOD1-G93A transgenic mouse model of amyotrophic lateral sclerosis. *J. Neuroimmune Pharmacol.* **2019**, *14*, 688–696. DOI: 10.1007/s11481-019-09866-x.
135. Zhou, Q.M. *et al*. n-butylidenephthalide treatment prolongs life span and attenuates motor neuron loss in SOD1G93A mouse model of amyotrophic lateral sclerosis. *CNS Neurosci. Ther.* **2017**, *23*, 375–385. DOI: 10.1111/cns.12681.
136. Zhu, Y. *et al*. All-trans retinoic acid exerts neuroprotective effects in amyotrophic lateral sclerosis-like Tg (SOD1*G93A)1Gur mice. *Mol. Neurobiol.* **2020**, *57*, 3603–3615. DOI: 10.1007/s12035-020-01973-8.
137. Zwiegers, P. *et al*. Reduction in hSOD1 copy number significantly impacts ALS phenotype presentation in G37R (line 29) mice: Implications for the assessment of putative therapeutic agents. *J. Negat. Results Biomed.* **2014**, *13*, 14. DOI: 10.1186/1477-5751-13-14.

Table S1. *List of references per category* - reporting experimental procedures and pathological mechanisms included in the categories used for metanalysis (please see main text). The reference numbers are referred to the publications listed above.

**Experimental procedures**	**References** (from the list of primary studies included in metanalysis)
Stress	1, 2, 10, 24, 33, 34, 40, 43, 47, 48, 50, 55, 93, 94, 99, 101, 103, 125, 126, 127, 129, 132, 135
Proteinopathy	11, 13, 28, 43, 56, 62, 64, 72, 89, 91, 92, 95, 112, 121, 128, 130
Immune response	4, 5, 6, 9, 10, 12, 13, 18, 27, 28, 30, 31, 46, 51, 80, 97, 101, 126, 127
Cell fate mechanisms	2, 3, 60, 77, 78, 90, 96, 100, 106, 111, 114, 133
Trophic support	7, 8, 15, 17, 19, 21, 23, 26, 35, 36, 49, 53, 54, 58, 66, 74, 81, 82, 83, 85, 88, 101, 104, 109, 115, 125, 127
**Pathological mechanisms**	**References** (from the list of primary studies included in metanalysis)
Immune response	5, 6 ,8, 12, 13, 18, 19, 21, 27, 28, 30, 31, 34, 46, 48, 49, 51, 54, 56, 64, 66, 72, 77, 80, 82, 82, 83, 88, 90, 95, 101, 103, 104, 106, 109, 114, 121, 126, 127, 129, 132, 135
Neuromuscular degeneration	1, 2, 3, 5, 6, 8, 10, 11, 12, 13, 15, 17, 19, 21, 23, 27, 28, 30, 33, 34, 36, 40, 43, 46, 48, 49, 54, 55, 56, 58, 60, 66, 72, 74, 77, 78, 80, 88, 89, 91, 92, 95, 99, 100, 101, 103, 104, 106, 109, 111, 114, 115, 121, 125, 126, 127, 128, 129, 130, 132, 133, 135
